# The effect of peer relationships on academic burnout among rural left-behind children: a chain mediating effect of social support and cognitive reappraisal

**DOI:** 10.3389/fpubh.2026.1789969

**Published:** 2026-07-13

**Authors:** Xiaofen Ding, Jun Tao, Yang Qian, Ziping Fan, Yipin Luo, Si Li, Weixin Dong, Chunxia Lu

**Affiliations:** 1 Hunan First Normal University, Changsha, Hunan, China; 2 Department of Sport Education, Hunan Normal University, Changsha, Hunan, China

**Keywords:** academic burnout, cognitive reappraisal, peer relationships, rural left-behind children, social support

## Abstract

**Objective:**

Despite growing evidence on the association between peer relationships and academic burnout among left-behind children, the underlying psychological mechanisms remain insufficiently understood. This study targeted rural left-behind children experiencing moderate to high academic burnout, and aimed to explore the serial mediating roles of social support and cognitive reappraisal in the relationship between peer relationships and academic burnout among this high-risk vulnerable group.

**Methods:**

This study employed a cross-sectional design, recruiting a total of 888 eligible rural left-behind children after excluding participants with low academic burnout as participants. All participants completed the Peer Relationship Scale, the Academic Burnout Scale, the Social Support Scale, and the Cognitive Reappraisal subscale of the Emotion Regulation Questionnaire. Data analysis was conducted using SPSS 27.0, Amos 29.0, and the PROCESS macro.

**Results:**

Regression analysis confirmed a significant negative correlational association between peer relationships and academic burnout (β = −0.325, *p* < 0.001). Further mediation analyses identified significant statistical correlational pathways linking these variables: this cross-sectional association was transmitted not only through the significant independent correlational pathway roles of social support [β = −0.132, 95% CI (−0.188, −0.078)] and cognitive reappraisal [β = −0.075, 95% CI (−0.116, −0.041)], but also, critically, through the correlational linkage of cognitive reappraisal specifically between social support and academic burnout. These pathways converged into a significant chain mediation correlational effect from social support to cognitive reappraisal [β = −0.022, 95% CI (−0.037, −0.009)].

**Conclusion:**

Among rural left-behind children with moderate-to-high academic burnout, peer relationships negatively affect academic burnout via the independent and serial mediating roles of social support and cognitive reappraisal, with social support exerting a stronger mediating effect.

## 1 Introduction

Amidst China's rapid urbanization and large-scale rural-to-urban migration, the psychological development of rural left-behind children (RLBC) has become an increasingly prominent concern. According to educational statistics released by the Ministry of Education in August 2023, the number of left-behind children at the compulsory education stage in rural areas was 15.5056 million (1). These children are typically defined as rural-registered minors under the age of sixteen, with both parents or one parent working as migrant laborers while the other lacks guardianship capacity, rendering them unable to live normally with their parents (2). This structural parental separation not only deprives children of the crucial emotional support and daily supervision essential for development but also places them at heightened risk for both psychosocial maladjustment and educational disadvantage (3). Within this context, academic burnout—a psychological syndrome characterized by emotional exhaustion, academic cynicism, and diminished personal accomplishment—emerges as a core manifestation of these risks in the educational and psychological domains (4). For left-behind children who chronically lack parental companionship and effective support, coping with academic pressures can more readily lead to feelings of isolation, helplessness, and emotional depletion (5), thereby catalyzing the onset of burnout. This condition not only directly undermines their academic engagement and performance but also, as a persistent state of psychological stress, serves as a significant risk factor for triggering more severe internalizing problems such as anxiety and depression (6). This adverse effect is particularly prominent among rural left-behind children with moderate-to-high academic burnout. Consequently, academic burnout is far from an isolated learning issue; rather, it constitutes a key nexus for understanding the overall mental health and adaptive challenges faced by left-behind children.

This pressing reality has galvanized a systematic policy response at the national level. Initiatives ranging from specific proposals at the 2025 National People's Congress advocating for “enhanced mental health services for vulnerable and left-behind children” (7) to the subsequent inclusion of “targeted care for special student groups” as a formal institutional requirement in the Ministry of Education's Ten Measures for Strengthening Mental Health Work in Primary and Secondary Schools underscore the urgency of establishing robust support systems (8). However, the effective translation of these macro-level policy directives into actionable school- and community-based interventions hinges on a precise, micro-level understanding of the underlying mechanisms of change. Given the structural deficit in familial emotional support commonly experienced by RLBC, peer relationships constitute their most immediate and accessible socio-emotional resource (9). The quality of these relationships is pivotal: positive peer interactions can serve as a crucial compensatory source of support, whereas negative experiences can exacerbate stress and alienation (10). Consequently, moving beyond broad correlational claims to precisely delineate the specific psychological mechanisms through which peer relationships influence academic burnout represents a fundamental and necessary research endeavor.

The present study specifically focuses on rural left-behind children with moderate-to-high academic burnout, aiming to explore the mediating roles of social support and cognitive reappraisal, as well as their independent and serial mediating mechanisms between peer relationships and academic burnout.

Against the unique developmental backdrop of RLBC with moderate-to-high academic burnout, this study conceptualizes social support through the lens of the Optimal Matching Theory of Social Support (11) and the Relational Flourishing Model (12), moving beyond the conventional conceptualization that reduces social support to a unidimensional construct of “objective assistance or emotional care provided by others” (13). Instead, social support is framed as a dynamic, interactive, and context-embedded interpersonal process (14), with its primary function being to facilitate adaptive adjustment and enhance subjective wellbeing among RLBC. From the perspective of the Optimal Matching Theory, the mediating role of social support is contingent upon the degree of congruence between expected social support (ESS) and actually perceived social support (ASS) (15), rather than the absolute quantity of support available. This theoretical proposition carries particular relevance to RLBC, a population that frequently turns to peer relationships to compensate for the lack of adequate family support. Building on this foundation, the Relational Flourishing Model further posits that social support exerts a dual, long-term mediating effect: it acts as a “buffer” that mitigates academic stress experienced by RLBC (16), while simultaneously serving as a “catalyst” that fosters the development of their positive cognitive tendencies. Integrating these theoretical frameworks, recent empirical scholarship has refined the multidimensional structure of social support by differentiating between subjective perceived support and objective behavioral support. These studies underscore that subjective perceived support—which is closely linked to the psychological adaptation of RLBC—plays a more salient mediating role in the association between peer relationships and such emotional-cognitive outcomes as academic burnout (17). That said, the specific mediating pathway through which social support operates in the link between peer relationships and academic burnout remains underexplored in the context of RLBC, a critical gap that the present study seeks to address.

Second, this study delves into the mediating mechanism of cognitive reappraisal from a micro-theoretical perspective, integrating the latest advances in Schema Theory (18) and Dual-System (19) Theory, along with their implications for individual differences. As an antecedent-focused emotion regulation strategy, cognitive reappraisal has moved beyond Gross's classic definition (20)—the process of modulating emotional responses by reinterpreting the personal meaning of emotion-eliciting events—to achieve new theoretical extensions. Drawing on the Schema Theory framework, cognitive reappraisal is essentially a process of “schema updating and reconstruction” (21): for RLBC who often hold negative cognitive schemas as a result of parental separation, this strategy can restructure their interpretations of academic stressors, thereby altering their emotional response patterns. Notably, this theoretical extension emphasizes that cognitive reappraisal is contingent on the interaction between positive peer feedback and individual cognitive resources—a proposition of particular relevance to RLBC (22), whose cognitive resources are often depleted due to chronic stress. The Dual-System Theory further refines the mechanism underlying individual differences: RLBC who maintain a flexible balance between the “automatic system” and the “controlled system” are more likely to deploy cognitive reappraisal effectively to cope with academic stress (23). Concurrently, as a critical external protective resource, social support not only provides RLBC with positive feedback cues to facilitate the updating of their cognitive schemas but also alleviates the depletion of cognitive resources (24). In doing so, it lays a foundational basis for the effective implementation of cognitive reappraisal strategies, enabling the modification of negative cognitive interpretations of academic stress. It is precisely on this basis that the present study identifies its core focus, undertaking a systematic and in-depth investigation to address the underdeveloped aspects of existing research.

Finally, in the context of RLBC with moderate-to-high academic burnout, to avoid isolated theoretical elaboration, this study incorporates the dual mediating roles of social support and cognitive reappraisal into an integrated theoretical framework. It is emphasized that positive peer relationships are hypothesized to enhance individuals perceived social support, which in turn supplements cognitive resources and provides secure environmental cues for the effective implementation of cognitive reappraisal strategies, thus forming a mutually reinforcing mechanism (25). Nevertheless, despite these theoretical underpinnings, two critical gaps persist in the existing literature. First, it remains underexplored whether social support and cognitive reappraisal function as independent parallel mediators in the association between peer relationships and academic burnout among RLBC. Second, drawing on the Broaden-and-Build Theory, the potential sequential resilience pathway—where supportive peer relationships first boost perceived social support, then facilitate the development of cognitive reappraisal ability, and ultimately mitigate academic burnout—has not yet been empirically validated.

**Figure 1:**
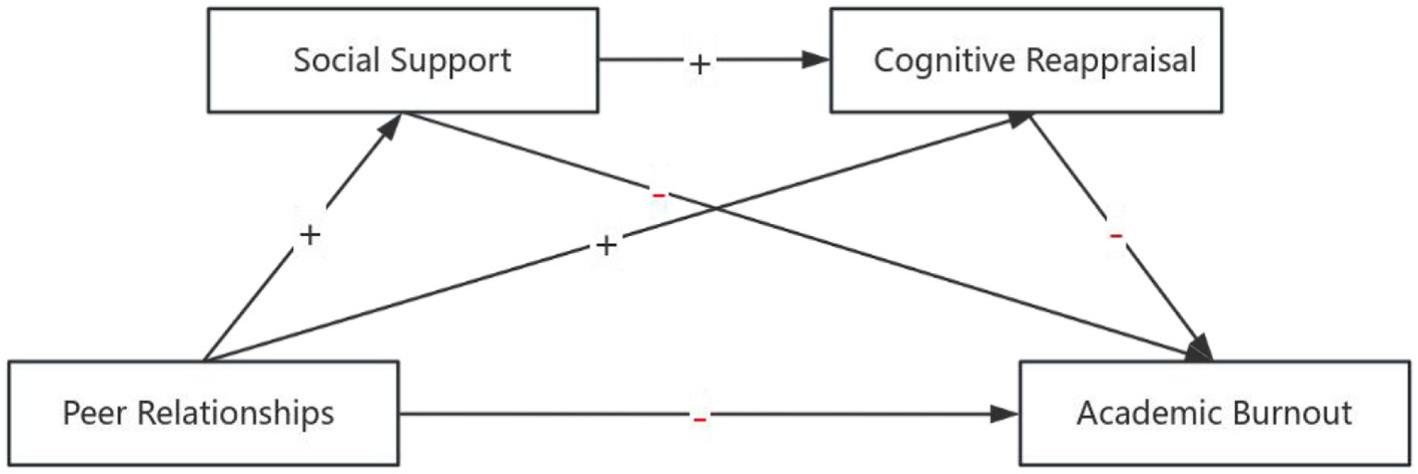
A hypothetical model of the influence of peer relationships and academic burnout.

Accordingly, this study aims to examine and verify the mechanistic gaps pertaining to the roles of social support and cognitive reappraisal in the relationship between peer relationships and academic burnout among RLBC with moderate-to-high academic burnout. The following hypotheses are proposed (the hypothesized model is shown in Figure 1):

**H**_**1**_**:** Peer relationships will significantly and negatively predict academic burnout.

**H**_**2**_**:** Social support will mediate the relationship between peer relationships and academic burnout.

**H**_**3**_**:** Cognitive reappraisal will mediate the relationship between peer relationships and academic burnout.

**H**_**4**_: Cognitive reappraisal will mediate the relationship between social support and academic burnout.

**H**_**5**_**:** Social support and cognitive reappraisal will act as serial mediators in the relationship between peer relationships and academic burnout.

## 2 Design

### 2.1 Participants and context

From October to December 2025, this study recruited left-behind children via a two-stage stratified cluster random sampling method from two rural public schools in Hunan Province, namely Helong School in Sangzhi County, Huaihua City, and Xinhang Middle School in Shaoyang City. Both schools implement a complete nine-year compulsory education system, which provides a solid guarantee for the comprehensiveness of the research sample. The selection of these two schools for the present study was based on rigorous considerations of their representativeness, typicality, and research applicability, with specific justifications as follows: First, as representative rural schools in Hunan Province, they can truly reflect the living and educational environments of the target population in this region. Second, the proportion of left-behind children in both schools is basically consistent with the average level of rural schools across the province; additionally, the two schools are highly comparable in terms of school-running conditions and structural characteristics, which effectively avoids sampling bias caused by extreme cases.

**Figure 2:**
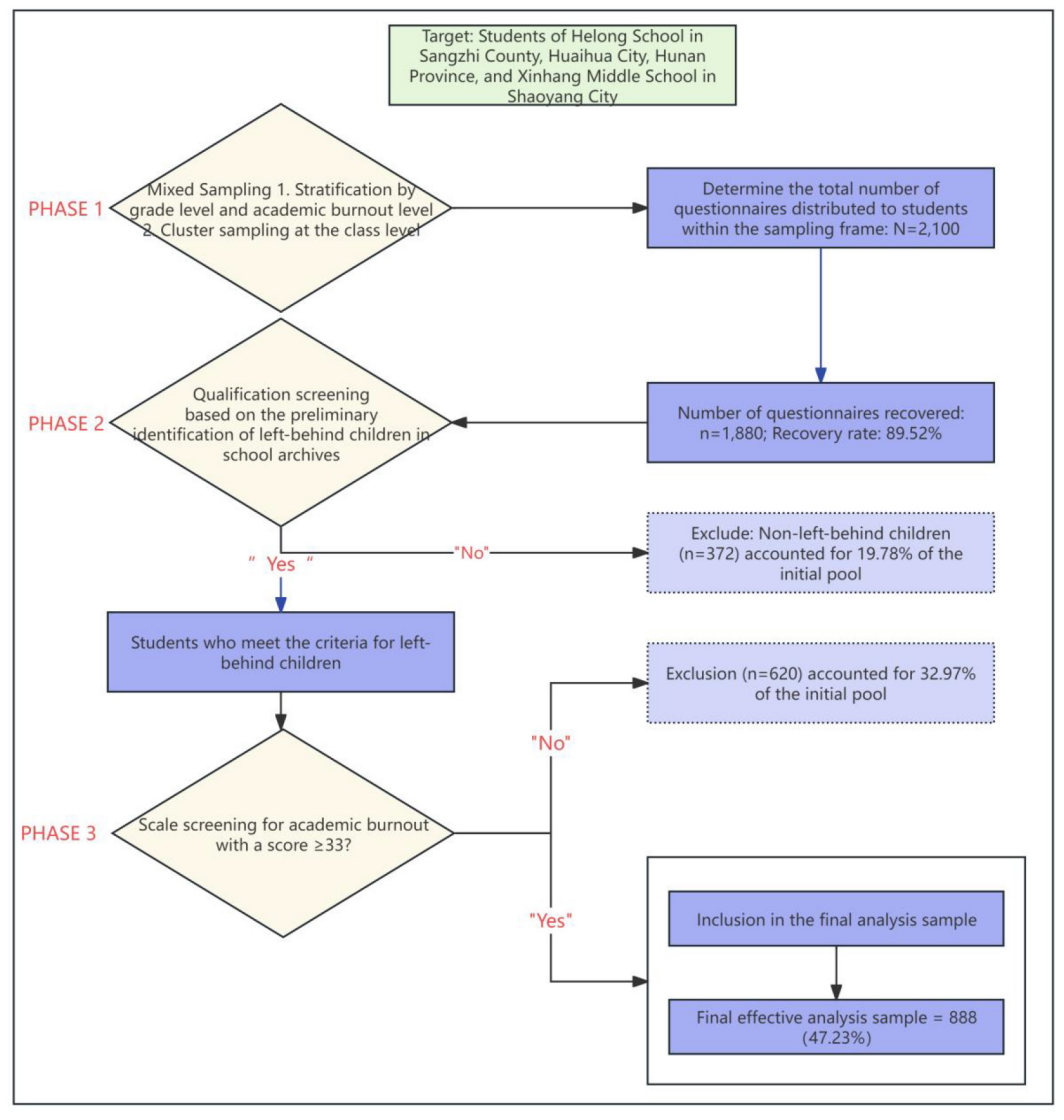
Recruitment process flow chart.

The sampling procedure was conducted in two stages. First, stratified random sampling was performed based on grade level (Grades 4–9) as the stratification variable. Second, cluster random sampling was applied at the class level to identify students who met the standardized definition of left-behind children. Prior to formal statistical analysis, data screening and quality control were strictly implemented following a standardized procedure. A total of 2,100 questionnaires were distributed within the predefined sampling frame, and 1,880 valid questionnaires were returned, resulting in a response rate of 89.52%. Notably, none of the returned questionnaires were excluded due to excessive missing values or problematic response patterns (*n* = 0), ensuring that all 1,880 returned questionnaires proceeded to the formal data quality control stage. Potential participants were identified through the school registration database. A total of 372 non-left-behind children were excluded from the initial sample, accounting for 19.78% of the screened population. Further screening was performed using the Chinese version of the Academic Burnout Scale. Consistent with the predefined cutoff criterion, participants with scores below the threshold of 33 were excluded (*n* = 620, accounting for 32.97% of the screened sample), with the aim of focusing on the target population at risk of academic burnout. However, this inclusion criterion may introduce selection bias: the final sample only comprises left-behind children with moderate to high academic burnout and lacks representativeness of the low-burnout subgroup. Consequently, the final analytical sample comprised 888 students, representing 47.23% of the screened population (see Figure 2).

The sample's gender distribution was 419 males (47.1%) and 469 females (52.9%). Age distribution was categorized into three groups: 9–11 years (*n* = 300, 33.7%), 12–14 years (*n* = 476, 53.6%), and 15–16 years (*n* = 112, 12.6%). Grade distribution was divided into two groups: grades 4–6 (*n* = 169, 19.0%) and grades 7–9 (*n* = 719, 81.0%). Regarding family structure, 115 students (12.9%) were only children, while 773 (87.0%) had siblings. In terms of parental marital status, the majority of the sample came from families with married parents (*n* = 681, 76.6%). The remaining distribution was as follows: divorced parents (*n* = 125, 14.0%), widowed parent (*n* = 17, 1.9%), and remarried parents (*n* = 65, 7.3%). As for guardianship, the sample was primarily categorized into three types: paternal grandparents (*n* = 428, 48.1%), maternal grandparents (*n* = 401, 45.1%), and other guardianship arrangements (*n* = 59, 6.6%). Finally, based on parental migration status, the sample composition was as follows: children with both parents away (*n* = 425, 47.8%), with only the father away (*n* = 350, 39.4%), and with only the mother away (*n* = 113, 12.7%). These demographic characteristics are summarized in Table 1.

**Table 1:** The demographics of the participants (*N* = 888).

Variables	Categories	Frequency	Percentage (%)
Gender	Male	419	47.1
	Female	469	52.9
Age	9–11	300	33.7
	12–14	476	53.6
	15–16	112	12.6
Grade	4–6	169	19.0
	7–9	719	81.0
Singleton status	Singleton	115	12.9
	Non-only-child	773	87.0
Parental marital status	Married	681	76.6
	Dissociaton	125	14.0
	Bereft of one's spouse	17	1.9
	Remarry	65	7.3
Type of guardian	Paternal grandparents	428	48.1
	Maternal grandparents	401	45.1
	other	59	6.6
Parental labor migration	Both parents	425	47.8
	Only the father	350	39.4
	Only the mother	113	12.7

### 2.2 Procedures

This study protocol received ethical approval from the Institutional Review Board of Hunan Normal University. Prior to formal data collection, all scales were developed, cross-culturally validated for the Chinese population, and further adapted for rural left-behind children. A pilot test with 55–65 rural left-behind children per scale examined item clarity. Pilot test psychometric properties confirmed reliability: internal consistency [Cronbach's α: 0.78–0.86, 95% CI (0.72, 0.90)] and structural validity [EFA: item factor loadings 0.62–0.89, cumulative variance explained 68.3%−75.6%, 95% CI (62.1%, 80.5%)]. To ensure the cultural fit, comprehensibility, and contextual appropriateness of the research items, a rigorous review was conducted by a panel consisting of psychology experts and rural teachers. This review process resulted solely in minor linguistic revisions, including substituting urban education terms with rural equivalents and streamlining expressions for younger student participants, while no major structural adjustments were implemented. Psychometric properties were further verified in the formal sample to establish validity for the target population.

Following these preparatory steps, the researchers provided full disclosure to all potential participants and their legal guardians regarding the study's aims, procedures, data confidentiality protocols, and potential risks. These risks included, but were not limited to, the possibility of transient emotional discomfort, the time commitment involved (approximately 30 min), and concerns regarding privacy. It was explicitly stated that all data would undergo strict anonymization through the use of unique identification codes, and that only aggregated, group-level analyses would be reported, with no personally identifiable information being disclosed. Upon full comprehension of this information, a written informed consent form was signed by the guardian of each participant. The participants themselves were also clearly informed of their right to withdraw unconditionally at any point during the questionnaire session without incurring any negative consequences. To ensure procedural integrity and participant wellbeing, the distribution, administration, and collection of questionnaires were directly supervised by the principal investigator in a standardized classroom setting to minimize fatigue. Trained research assistants and a school psychologist were present throughout the session to provide immediate emotional support and guidance should any participant experience reflection-related or questionnaire-induced discomfort. Prior to commencing their duties, all personnel involved in data collection completed a unified training program on standardized operating procedures and research ethics, with specific instruction on recognizing and appropriately responding to potential mild emotional reactions from participants. Finally, all collected data were entered into an electronic database via double independent entry and cross-verified to ensure accuracy.

### 2.3 Methods

#### 2.3.1 Peer relationships scale

The Peer Relationships Scale (26), consisting of 30 items, was used to assess peer relationships. The scale comprises two subscales: Peer Acceptance (20 items; 6 positively worded, 14 reverse-scored, all recoded) and Peer Fear/Inferiority (10 items; all positively worded). Responses were recorded on a 4-point Likert scale (1 = “Completely Disagree,” 4 = “Completely Agree”). Higher scores on the Peer Acceptance subscale indicate better perceived peer relationships, whereas higher scores on the Peer Fear/Iniority subscale indicate greater social anxiety and poorer peer relationships. This scale is suitable for evaluating the subjective social experiences of rural left-behind children. In the current study sample, the scale exhibited outstanding psychometric characteristics, with relevant details presented in Table 2 below.

**Table 2:** Psychometric properties of each scale.

Scale	Cronbach's α (95%CI)	KMO	Bartlett's test of sphericity			Average factor loading
			χ^2^	df	*p*	
Peer relationships scale	0.905 [95% CI = (0.770, 0.962)]	0.943	13,139.955	435	< 0.001	0.535
Academic burnout scale	0.911 [95% CI = (0.898, 0.925)]	0.937	8,306.901	210	< 0.001	0.699
Cognitive reappraisal scale	0.931 [95% CI = (0.926, 0.937)]	0.922	4,015.158	15	< 0.001	0.823
Social support scale	0.954 [95% CI = (0.930, 0.979)]	0.946	10,509.907	66	< 0.001	0.780

#### 2.3.2 Academic burnout scale

This study employed the Chinese version of the Academic Burnout Scale developed by Hu and Dai (27). This scale uses a 5-point Likert scale, with scores ranging from 1 to 5. The questionnaire consists of 21 items, divided into four dimensions: Emotional Exhaustion (8 items), Alienation from Teachers (4 items), Physical Exhaustion (4 items), and Reduced Learning Efficacy (5 items, reverse-scored and subsequently recoded positively). Based on the total score, participants were categorized into a “high burnout group” (≥68) and a “low burnout group” (< 33). In the present study, participants with low academic burnout were excluded. Methodologically, this study aims to investigate the mechanism of academic burnout, and low-burnout individuals lack typical burnout characteristics. Retaining these samples would weaken the statistical efficiency of correlation and mediating effect analyses. Hence, we enrolled only rural left-behind children with moderate-to-high academic burnout as research subjects. Prior to administration, all items were reviewed by experts and rural teachers to ensure cultural and linguistic appropriateness for the target population, thereby minimizing contextual misinterpretations. In the present sample, the scale demonstrated excellent psychometric properties. Detailed information is provided in Table 2 below.

#### 2.3.3 Social support scale

The Chinese version of the Perceived Social Support Scale (PSSS) was employed in this study. The scale was originally developed by Blumenthal et al. in 1987 and was later translated and adapted into Chinese by Huang Li et al. (28). It consists of 12 items measuring three dimensions of support: from family, from friends, and from significant others. The total score ranges from 12 to 84, with scores of 12–36 indicating low support, 37–60 indicating moderate support, and 61–84 indicating high support. Excellent psychometric properties were observed for the scale within the present sample, with comprehensive data shown in Table 2.

#### 2.3.4 Cognitive reappraisal scale

Cognitive reappraisal was assessed using the Cognitive Reappraisal subscale of the Emotion Regulation Questionnaire (ERQ) developed by Gross and John (29). This subscale consists of 6 items designed to measure the habitual use of reappraisal to alter the emotional impact of a situation (e.g., “When I want to feel less negative emotion, I change the way I'm thinking about the situation”). Participants rated each item on a 5-point Likert scale ranging from 1 (“strongly disagree”) to 5 (“strongly agree”). A mean score was computed across the six items, with higher scores indicating a greater tendency to employ cognitive reappraisal. The scale used in this study showed remarkable psychometric properties among the selected sample; corresponding details are listed in Table 2 below.

### 2.4 Data analysis

The data processing and analysis in this study adhered to standard methodological procedures. First, collected questionnaires were screened to exclude invalid responses with obvious patterned answering or internal inconsistencies, and the valid data were coded and entered. Preliminary data screening was then conducted: outliers were identified via Mahalanobis distance and Z-scores (|Z| > 3.29) and retained after manual verification; Shapiro-Wilk tests and skewness/kurtosis values (ranging from −2 to +2) confirmed the normality assumption; and Little's MCAR test verified that missing data were completely at random, which were subsequently addressed using multiple imputation to ensure statistical robustness. Descriptive statistics and Pearson correlation coefficients were computed using SPSS 27.0. The measurement model was evaluated in AMOS 29.0, and upon confirming adequate model fit, a chain mediation model was constructed to test the study's hypotheses. The serial mediating effects of social support and cognitive reappraisal were examined using the bias-corrected non-parametric percentile bootstrap method with 5,000 resamples. Effects were deemed significant if the 95% confidence interval excluded zero. Significance levels are denoted throughout as ^*^p < 0.05, ^**^p < 0.01, and ^***^p < 0.001.

## 3 Results

### 3.1 Common method bias test

Since all data were collected via single-time-point self-report questionnaires, potential common method bias (CMB) existed. We first conducted Harman's single-factor test (30). Eleven factors with eigenvalues greater than 1 were extracted, and the first factor explained only 21.33% of the total variance, far below the 40% threshold, suggesting no severe CMB. We further adopted the unmeasured latent common method factor (UMCF) approach (31). The baseline measurement model exhibited good fit (χ^2^*/df* = 1.852, *df* = 431, CFI = 0.941, RMSEA = 0.047), with standardized item loadings ranging from 0.583 to 0.847. After controlling for common method variance, all item loadings on theoretical constructs remained significant at *p* < 0.001 (0.567–0.832). The fit changes were trivial (ΔCFI = 0.006, ΔRMSEA = −0.003), both below the 0.01 cutoff, indicating negligible improvement in model fit after incorporating the method factor. These results demonstrate that CMB is unlikely to fully account for our research findings. However, given the cross-sectional self-report design, minor CMB effects cannot be completely excluded.

### 3.2 Correlation analyses

**Figure 3:**
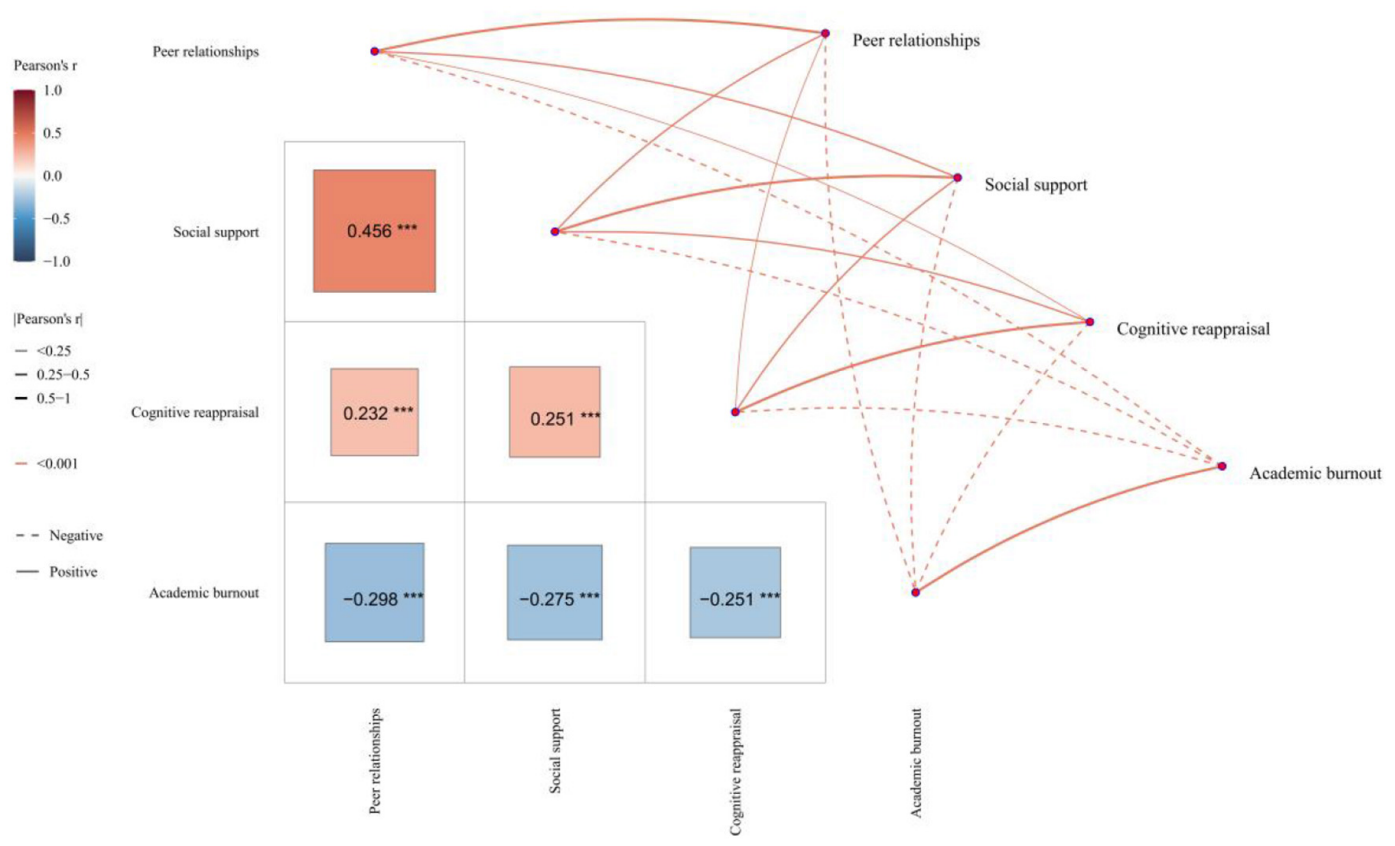
Mantel test of research variables. ^*^P < 0.05, ^**^P < 0.01, ^***^P < 0.001, significant differences.

All analyses in this study were restricted to rural left-behind children with moderate-to-high academic burnout after excluding participants with low academic burnout based on methodological rationality. Table 3 and Figure 3 present the bivariate correlations among peer relationships, academic burnout, social support, and cognitive reappraisal. To focus on the correlation among the core variables of the study and avoid the statistical interference of high correlation between sub-dimensions of the scale, we only calculated the Pearson correlation coefficients of the total scores of peer relationships, social support, cognitive reappraisal and academic burnout for the correlation analysis. Table 3 and Figure 3 present the bivariate correlations among peer relationships, academic burnout, social support, and cognitive reappraisal. Peer relationships was positive correlated with social support (*r* = 0.456, *p* < 0.01) and demonstrated a strong negative association with academic burnout (*r* = −0.298, *p* < 0.01). A positive correlation was observed between peer relationships and cognitive reappraisal (*r* = 0.232, *p* < 0.01). In contrast, social support and academic burnout showed divergent associations with cognitive reappraisal: a positive correlation for social support (*r* = 0.251, *p* < .01) and a negative correlation for academic burnout (*r* = −0.251, *p* < .01). Additionally, social support was negatively correlated with academic burnout. Supporting these findings, regression analysis in Table 4 identified peer relationships as a significant negative predictor of academic burnout (β = −0.325, *p* < .001).All subsequent results are presented as standardized values.

**Table 3:** Correlation coefficient matrix of research variables.

Variables	PR	SS	CR	AB
PR	1			
SS	0.456^**^	1		
CR	0.232^**^	0.251^**^	1	
AB	−0.298^**^	−0.275^**^	−0.251^**^	1

PR, Peer relationships; SS, Social support; CR, Cognitive Reappraisal; AB, Academic Burnout; all reported coefficients are standardized. ^**^P < 0.01, significant differences.

**Table 4:** Cognitive reappraisal bootstrap mediation effect tests.

Path	Effect size	Boot SE	*t*	*p*	Bias-corrected 95% CI		Effect ratio
					Lower	Upper	
Total effect	−0.271	0.031	−8.544	< 0.001^***^	−0.334	−0.209	–
Direct effect	−0.224	0.032	−6.967	< 0.001^***^	−0.287	−0.161	82.66
Indirect effect	−0.047	0.012	_−_	< 0.001^***^	−0.071	−0.027	17.34

All reported coefficients are standardized. ^***^P < 0.001, significant differences.

### 3.3 Bootstrap analysis of mediating effect significance test

#### 3.3.1 Mediating role of social support between the peer relationships and academic burnout

Bootstrap analysis provided support for the significant mediating role of social support. The indirect effect of peer relationships on academic burnout through social support was statistically significant [indirect effect = −0.132, 95% Boot CI (−0.188, −0.078)], and the direct effect remained significant [direct effect = −0.369, 95% Boot CI (−0.484, −0.254)] Given that neither confidence interval contained zero, both the indirect and direct effects were statistically robust.

**Figure 4:**
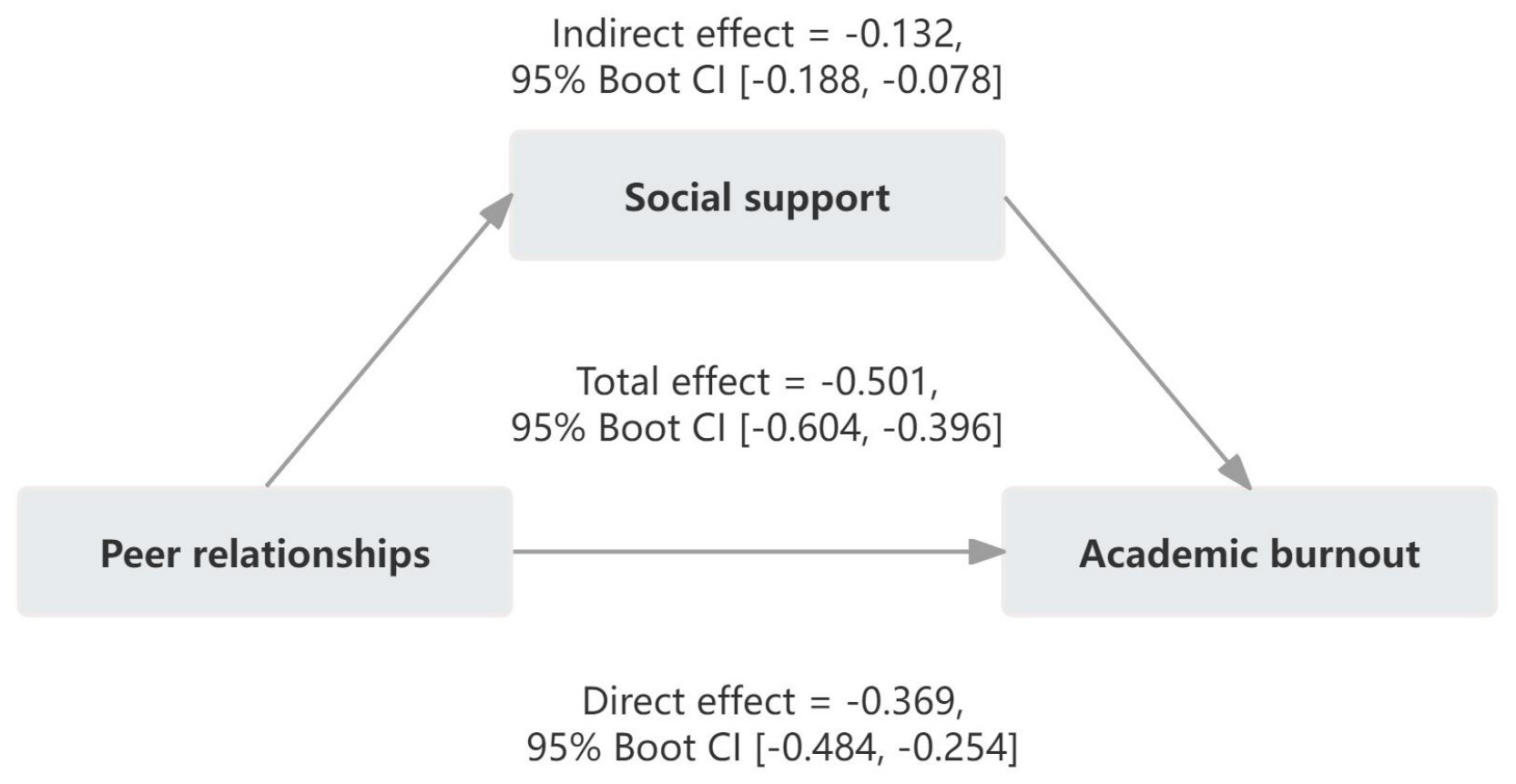
The mediation analysis model of social support.

To further characterize the magnitude and practical significance of the mediating pathway, the effect size corresponding to the mediated proportion was computed as the ratio of the indirect effect to the total effect, yielding a value of 26.35%. This effect size demonstrates that social support accounted for approximately one-quarter of the total effect of peer relationships on academic burnout, indicating a moderate, practically meaningful mediating effect beyond mere statistical significance. This magnitude suggests that higher quality peer relationships may diminish academic burnout both directly and indirectly by fostering greater social support, with meaningful implications for intervention design and preventive practice. Taken together, these findings support the conclusion that social support functions as a partial mediator in the association between peer relationships and academic burnout. Detailed results are presented in Table 5 and Figure 4.

**Table 5:** Social support bootstrap mediation effect tests.

Path	Effect size	Boot SE	*t*	*p*	Bias-corrected 95% CI		Effect ratio
					Lower	Upper	
Total effect	−0.501	0.053	−9.446	< 0.001^***^	−0.604	−0.396	_−_
Direct effect	−0.369	0.059	−6.272	< 0.001^***^	−0.484	−0.254	73.65
Indirect effect	−0.132	0.028	_−_	< 0.001^***^	−0.188	−0.078	26.35

All reported coefficients are standardized. ^***^P < 0.001, significant differences.

**Table 6:** Cognitive reappraisal bootstrap mediation effect tests.

Path	Effect size	Boot SE	*t*	*p*	Bias-corrected 95% CI		Effect ratio
					Lower	Upper	
Total effect	−0.501	0.053	−9.446	< 0.001^***^	−0.605	−0.397	–
Direct effect	−0.426	0.054	−7.934	< 0.001^***^	−0.531	−0.320	85.03
Indirect effect	−0.075	0.019	–	< 0.001^***^	−0.116	−0.041	14.97

All reported coefficients are standardized. ^***^P < 0.001, significant differences.

#### 3.3.2 Mediating role of cognitive reappraisal between the peer relationships and academic burnout

Bootstrap analyses revealed that cognitive reappraisal significantly mediates the association between peer relationships and academic burnout. The indirect effect was statistically significant [indirect effect = −0.075, 95% CI (−0.116, −0.041)], and the direct effect remained significant [direct effect = −0.426, 95% CI (−0.531, −0.320)]. Importantly, neither confidence interval contained zero, providing evidence for the statistical reliability and robustness of both direct and indirect pathways.

**Figure 5:**
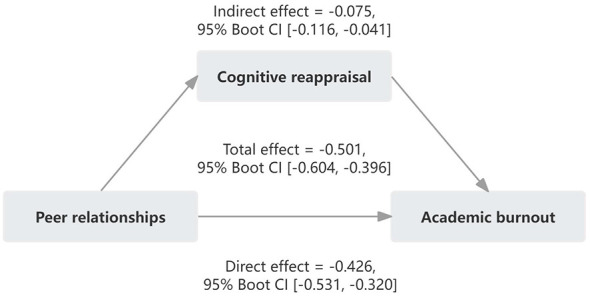
The mediation analysis model of cognitive reappraisal.

To further clarify the magnitude and practical significance of this mediating mechanism, the proportion of the total effect mediated by cognitive reappraisal was estimated at 14.97%. This effect size indicates that cognitive reappraisal serves as a substantive yet partial mediator in the link between peer relationships and academic burnout, explaining a meaningful proportion of the observed association beyond statistical significance. Taken together, these findings highlight cognitive reappraisal as a meaningful explanatory mechanism underlying the association between peer relationships and academic burnout. Detailed results are presented in Table 4 and Figure 5.

#### 3.3.3 Mediating role of cognitive reappraisal between the social support and academic burnout

Bootstrap analyses indicated that cognitive reappraisal significantly mediates the association between social support and academic burnout. The indirect effect was statistically significant [indirect effect = −0.047, 95% CI (−0.071, −0.027)], and the direct effect remained significant [direct effect = −0.224, 95% CI (−0.287, −0.161)]. Neither confidence interval contained zero, thereby supporting the statistical robustness of both direct and indirect pathways.

**Figure 6:**
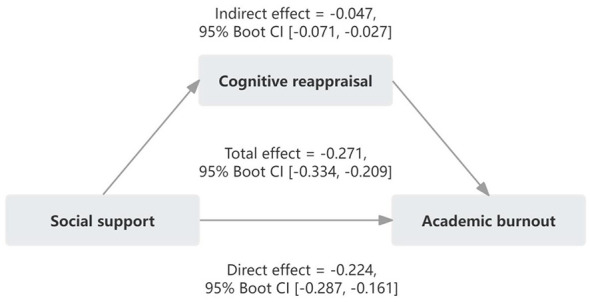
The mediation analysis model of social support.

To further demonstrate the magnitude and practical significance of this mediating pathway, the proportion of the total effect explained by cognitive reappraisal was estimated at 17.34%. This effect size suggests that cognitive reappraisal represents a substantive yet partial mediator in the relationship between social support and academic burnout, providing a meaningful explanatory mechanism beyond statistical significance. Collectively, these findings underscore the role of cognitive reappraisal in linking social support to reduced academic burnout. Detailed results are presented in Table 4 and Figure 6.

#### 3.3.4 Mediating role of social support and cognitive reappraisal between the peer relationships and academic burnout

**Figure 7:**
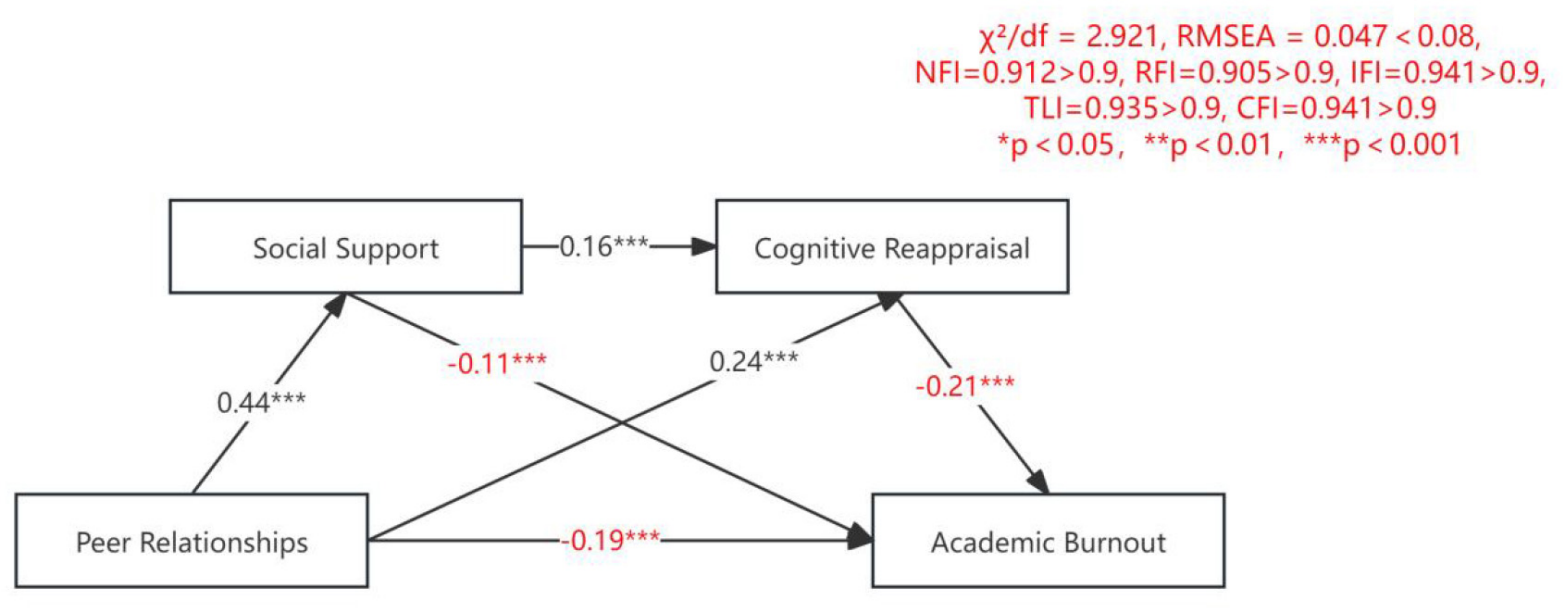
A chain-mediated model. ^*^P < 0.05, ^**^P < 0.01, ^***^P < 0.001, significant differences.

To test the proposed chain mediation model in which social support and cognitive reappraisal sequentially mediate the association between peer relationships and academic burnout, we first evaluated the overall structural model. The model exhibitedexcellent model fit: χ^2^*/df* = 2.921, RMSEA = 0.047, NFI = 0.912, RFI = 0.905, IFI = 0.941, TLI = 0.935, and CFI = 0.941. As illustrated in Figure 7, all hypothesized structural paths were statistically significant (*p* < 0.05). Specifically, peer relationships were positively associated with both social support (β = 0.769) and cognitive reappraisal (β = 0.103). After accounting for the mediating roles of social support and cognitive reappraisal, peer relationships still exerted a significant direct negative effect on academic burnout (β = −0.325), indicating the presence of both direct and indirect pathways (see Tables 7, 8).

**Table 7:** Regression analysis of the relationship between peer relationships, social support, cognitive reappraisal and academic burnout.

Variables	Characteristics	Adj R-sq	*F*	Beta	*t*
Social support	Gender	0.210	78.374^***^	0.122	0.127
	Age			−0.347	−1.519
	Peer relationships			0.769	15.283
Cognitive reappraisal	Gender	0.119	30.076^***^	0.127	0.324
	Age			−0.586	−6.293
	Social support			0.066	4.825
	Peer relationships			0.103	4.484
Academic burnout	Gender	0.146	30.252^***^	2.562	2.593
	Age			0.067	0.279
	Cognitive reappraisal			−0.430	−5.078
	Social support			−0.143	−4.085
	Peer relationships			−0.325	−5.530

All reported coefficients are standardized. ^***^P < 0.001, significant differences.

**Table 8:** Test of the chain mediation model.

Path	Effect size	BootSE	*t*	*p*	Bias-corrected 95% CI		Effect ratio
					Lower	Upper	
Total effect	−0.501	0.053	−9.446	< 0.001^***^	−0.604	−0.392	–
Direct effect	−0.325	0.059	−5.530	< 0.001^***^	−0.439	−0.209	64.87
Indirect effect	−0.176	0.031	–	< 0.001^***^	−0.238	−0.117	35.13
PR → SS → AB	−0.109	0.028	–	< 0.001^***^	−0.164	−0.055	21.75
PR → CR → AB	−0.044	0.016	–	< 0.001^***^	−0.079	−0.018	8.78
PR → SS → CR → AB	−0.022	0.007	–	< 0.001^***^	−0.037	−0.009	4.39

All reported coefficients are standardized. ^***^P < 0.001, significant differences.

We subsequently conducted formal mediation tests using Hayes' PROCESS macro (Model 6) with 5,000 bootstrap resamples, controlling for gender and age, to verify the specific serial mediation pathway: peer relationships → social support → cognitive reappraisal → academic burnout. The total indirect effect was statistically significant [β = −0.176, Boot SE = 0.031, 95% CI (−0.238, −0.117)]. Importantly, the hypothesized serial mediation path was also significant [β = −0.022, Boot SE = 0.007, 95% CI (−0.037, −0.009)], supporting the sequential transmission of effects through social support and cognitive reappraisal. These results were further validated by standardized effect size analysis, which revealed a significant standardized indirect effect (β = −0.013; see Table 9). Collectively, these findings provide consistent and robust empirical support for the proposed chain mediation model, highlighting the sequential roles of social support and cognitive reappraisal in explaining how peer relationships are linked to lower levels of academic burnout among the targeted subgroup of rural left-behind children with moderate-to-high academic burnout.

**Table 9:** Standardization effect.

Effect type	Path	Standardization effect
Single mediation effect	PR → SS → AB	−0.066
Single mediation effect	PR → CR → AB	−0.026
Chain mediation effect	PR → SS → CR → AB	−0.013
Total indirect effect	PR → AB	−0.106

All reported coefficients are standardized.

## 4 Discussion

By constructing an integrated chain mediation model, this study systematically organizes and further elaborates on the scattered empirical findings regarding peer relationships and academic burnout in existing literature. It explicitly reveals the sequential mechanism through which peer relationships protect rural left-behind children with moderate-to-high academic burnout from academic burnout via psychological mediating pathways. All research hypotheses were fully verified. The results fill the critical gap in the academic community's understanding of this underlying mechanism.

This study confirms the direct protective effect of peer relationships on academic burnout (H1), further verifying the replicability and applicability of this core association in the unique group of rural left-behind children with moderate-to-high academic burnout (32). Beyond this direct effect, the findings on independent mediating roles (H2, H3) are grounded in previous isolated research achievements. Prior studies have identified social support and cognitive reappraisal as individual mediating variables between social relationships and burnout, respectively, yet few have integrated both into a unified research framework for comprehensive analysis (33). This study synthesizes these fragmented findings, confirming that peer relationships exert protective effects through two parallel pathways: the external resource supply pathway (social support) and the internal cognitive regulation pathway (cognitive reappraisal), thereby enriching the systematic understanding of the mechanisms underlying their functions.

Meanwhile, this study validates the sequential chain mediating pathways (H4, H5), a finding that directly addresses the core limitation of prior research. Previous studies either examined single mediating variables in isolation or treated multiple mediating variables as parallel mechanisms, failing to uncover their intrinsic connections. Unlike the single-mediator designs commonly adopted in existing studies on left-behind children, which tend to hypothesize simple direct or parallel mediating effects, this study explicitly demonstrates that the protective effect of peer relationships follows a structured sequential logic of resource empowerment → cognitive transformation. This finding echoes the recent social-cognitive resilience framework (34) and further extends the theoretical boundary of its application. This framework has criticized the obvious inadequacy of traditional models in capturing the progressive nature of psychological development among vulnerable adolescents, and the sequential mechanism identified in this study precisely compensates for this deficiency, providing clear theoretical support and contextual interpretation for the research results. Specifically, social support emerges as a stronger and dominant independent mediating variable (35), which is highly consistent with the basic needs satisfaction perspective in left-behind children research. For rural left-behind children with moderate-to-high academic burnout who have long been confronted with parental absence and emotional deprivation, peer relationships primarily serve as an immediate channel for them to obtain emotional support and a sense of belonging (36). This core function can directly diminish the loneliness caused by family separation and chronic academic stress (37), rendering social support a fundamental and most influential mediating link connecting peer relationships and academic burnout. This result profoundly reflects the unique developmental needs of this group: the role of external relational resources is more prominent than that of internal cognitive strategies. Furthermore, social support can further catalyze the process of cognitive restructuring (38), forming a critical chain pathway of social support → cognitive restructuring that underpins the ultimate protective effect of peer relationships. This finding breaks through the limitations of previous research, which mostly regarded social support and cognitive restructuring as independent predictors of occupational burnout without revealing the intrinsic association between them. The internal mechanism of this sequential relationship can be explained by the resource priming effect: left-behind children with reliable peer support are more likely to gain the psychological security required to adopt adaptive cognitive strategies. When children perceive stable emotional support, they tend to reinterpret academic stress as manageable challenges rather than overwhelming threats (39), thereby effectively alleviating academic burnout at the cognitive level.

In addition, this study interprets the relatively weak independent mediating effect of cognitive reappraisal. Unlike many previous mediation studies that only report non-significant or weak indirect effects, this result is theoretically interpreted as highlighting the unique challenges faced by rural left-behind children with moderate-to-high academic burnout: against the backdrop of resource scarcity and chronic stress, the process of transforming obtained social support into active and proficient cognitive regulation skills itself constitutes a critical transformation bottleneck (40). This implies that intervention measures that only focus on the supply side of fostering a friendly peer atmosphere will yield limited results if they neglect the transformation side of cultivating individuals' ability to utilize these resources for self-regulation. This insight directly responds to the academic call for more mechanism-based intervention studies instead of descriptive or correlational research, shifting the focus of intervention from what to provide to how to facilitate transformation. Meanwhile, in the traditional rural community culture that emphasizes collective mutual assistance, peer support networks often represent a potential, naturally formed resource (41). However, due to children's deficiencies in emotional regulation skills, the effectiveness of these resources may remain locked (42). Most previous studies have not explicitly explored how to activate these potential resources among left-behind children samples. The chain model proposed in this study provides a key pathway for unlocking this potential: intervention measures must adhere to the sequential principle. First, it is necessary to systematically activate and construct support networks, such as through peer mutual-aid groups. On this basis, micro-training on cognitive restructuring skills should then be embedded, for example, guiding stress restructuring during group activities. This intervention logic of establishing a support foundation first, then cultivating regulation skills not only aligns with the underlying mechanism but also maximizes the intervention effect.

Finally, while affirming the consistency and extensiveness of this study with existing academic achievements, it is also necessary to pay attention to the subtle differences between the results of this study and previous relevant research. These differences are not accidental but arise from the combined effects of multiple interrelated factors. First, previous studies on the association between peer relationships and academic burnout among left-behind children have often relied on small-scale convenience samples or focused on specific age groups (42). In contrast, this study adopts a large representative sample covering multiple grades in rural areas, and the differences in sample representativeness and diversity may lead to variations in the strength of mediating effects. For instance, the dominant role of social support observed in this study may be more pronounced in samples with a broader age range—because younger left-behind children are more dependent on external emotional support, whereas older children may engage in more complex cognitive regulation processes. Such age-specific dynamics may not have been fully captured in studies limited to a single age group. Second, previous studies may have concentrated on economically relatively developed rural areas (42), while this study covers underdeveloped rural areas with resource scarcity, where peer relationships serve as the primary source of social support. This contextual difference may amplify the mediating role of social support and highlight the transition bottleneck effect of cognitive reappraisal—children in resource-scarce environments have fewer opportunities to independently develop cognitive regulation skills. Subsequently, based on the findings from cross-sectional studies, a 1–2 year follow-up of the same cohort of rural left-behind children can be conducted, utilizing the Cross-Lagged Panel Model (CLPM) to verify the directionality of causal relationships between variables and the stability of sequential mediating paths. Finally, this study employs a revised scale specifically validated for rural left-behind children, whereas previous studies have frequently used general tools that may not fully capture the unique characteristics of peer relationships in rural settings, thus leading to underestimation or overestimation of effect sizes. Clarifying the causes of these differences can not only facilitate a more comprehensive interpretation of the results of this study but also provide targeted references for the design and implementation of subsequent related research.

In conclusion, this study transcends the general conclusion that peer relationships are beneficial—a conclusion that has dominated most existing literature on the mental health of left-behind children. By dissecting the chain mechanism of social support and cognitive restructuring among rural left-behind children with moderate-to-high academic burnout, it reveals the specific conditions and transformation bottlenecks that influence their effects. This analysis not only resolves the contradictions in previous studies through contextualized effect sizes and mediating roles but also promotes a paradigm shift in future research and practice—from simply promoting relationships to deliberately designing transformation processes. This sequential research framework not only confirms the significant value of positive peer interactions for rural left-behind children but also fills the gap in the field of resilience research by clarifying how external relational resources can be transformed into adaptive internal capabilities for this vulnerable group, thereby providing new ideas and theoretical references for subsequent related research.

## 5 Limitations and future research directions

### 5.1 Limitations

This study has several limitations. First, the cross-sectional research design imposes critical constraints on causal inference. This design only captures the data of variables at a single time point (October–December 2025), and cannot track the dynamic changes of peer relationships and academic burnout with the growth of left-behind children. Thus, it is impossible to verify the directionality of causality in the proposed serial mediation model, and the conclusion of “peer relationships alleviate academic burnout” is only a reasonable inference based on theoretical and cross-sectional correlational evidence. Second, the burnout-based inclusion criterion caused selection bias by excluding 620 low-scoring participants (< 33), leaving a sample of only moderate-to-high burnout cases. Thus, the findings are not generalizable to all rural left-behind children, especially those with low burnout. Third, the over-reliance on self-reported data threatens validity mainly through common method bias (CMB) and response bias. Future research should integrate multi-source data to cross-validate results and mitigate these biases. Fourth, the sample of this study was drawn exclusively from two counties in China, which constrains the external validity of the research findings. Given the high homogeneity of participants in terms of their cultural backgrounds, socioeconomic statuses, and educational experiences, the generalizability of these results to left-behind children in other regions or countries remains to be further verified. Finally, this study only examined the mediating roles of social support and cognitive reappraisal while ignoring other potential mechanisms and moderating factors. This results in an incomplete understanding of the underlying mechanisms of academic burnout. Subsequent research should construct an integrated model incorporating multiple mediating and moderating variables to explore these complex relationships.

### 5.2 Implications of this research

This research translates the social support → cognitive reappraisal pathway into actionable intervention principles targeting rural left-behind children with moderate to high academic burnout, and adjustments are required when applying these principles to those with low burnout levels. It shows that alleviating academic burnout in left-behind children requires more than vague peer relationship promotion; effective interventions must align with the underlying psychological mechanism: first, structurally strengthen their peer support networks to build a reliable socio-emotional scaffold, then embed targeted cognitive reappraisal training on this basis. Specifically, interventions to enhance peer support and cognitive reappraisal should follow these steps: teachers first assess students' peer support via validated scales, establish structured systems such as peer tutoring groups, and track and consolidate peer connections; they then teach age-appropriate academic and emotional stress reappraisal methods, reinforced through role-play, scenario exercises, daily tasks, and peer feedback; parents proactively understand their children's peer interactions, encourage participation in interactive activities, and guide them to apply cognitive reappraisal when stressed; students actively engage in peer mutual-aid activities, build stable relationships through sincere assistance, and fully participate in training and feedback exchanges. This foundation first support, core second regulation sequence, derived from the validated chain mediation model, ensures interventions precisely activate social support and cognitive reappraisal, systematically building a defense against burnout. Future research should adopt a 3-wave 1–2 year longitudinal design for rural left-behind children across low, moderate, and high academic burnout levels, using stratified sampling to ensure group representativeness, and employ CLPM to verify variable causality, pathway stability, and cross-group differences in the mediation pathway, thereby refining the proposed intervention principles.

## 6 Conclusions

The present study verifies that peer relationships reduce academic burnout among rural left-behind children with moderate-to-high academic burnout via the serial mediating pathway of social support and cognitive reappraisal. This finding suggests that effective interventions should follow the sequential logic of “first establishing support, then cultivating skills” for rural left-behind children with moderate to high academic burnout. Specifically, structured activities (e.g., peer support groups) should first be used to consolidate children's social support networks. Subsequently, cognitive reappraisal training should be embedded within this secure foundation. Therefore, school and policy designs should move beyond generalized care and instead support such evidence-based, sequential, and targeted intervention programs. In doing so, peer resource systems can be systematically transformed into psychological scaffolds that promote child development.

## Data Availability

The original contributions presented in the study are included in the article/supplementary material, further inquiries can be directed to the corresponding authors.
